# Neuroendocrine Carcinoma of the Uterine Cervix With Metastases to the Thyroid Gland: A Case Report and Clinical Pathological Review

**DOI:** 10.7759/cureus.29564

**Published:** 2022-09-25

**Authors:** Wendolin J Ortiz, Miriam A Gutierrez, Herman Mabrie, Mario Cervantes

**Affiliations:** 1 Pathology, Universidad Autónoma de Baja California, Mexicali, MEX; 2 Pathology, Universidad Xochicalco, Mexicali, MEX; 3 Otolaryngology - Head and Neck Surgery, United Memorial Medical Center, Houston, USA; 4 Pathology, United Memorial Medical Center, Houston, USA; 5 Pathology, HCA Houston Healthcare West and Pearland, Houston, USA

**Keywords:** fine-needle aspiration, immunohistochemistry, uterine cervix, metastatic neuroendocrine carcinoma, thyroid gland

## Abstract

Thyroid metastasis from a neuroendocrine carcinoma (NEC) is atypical, and the most common site of origin is the lung. We present the case of a 48-year-old lady with a history of NEC in the uterine cervix, classified initially as a p16-positive high-grade endocervical adenocarcinoma with endometrioid differentiation in a cervical biopsy. The patient, after having a thyroid ultrasound due to thyroid nodules, showing a multinodular goiter and suspicious nodules, and a subsequent fine-needle aspiration with a diagnosis of papillary thyroid carcinoma, presented to our hospital for a total thyroidectomy. Histologically, there were metastatic high-grade carcinoma foci within the thyroid, consistent with metastasis from the cervical primary tumor based on the morphology and immunohistochemical stains, the tumor was re-classified as an NEC. The thyroid gland is an uncommon site for metastasis from primary sites, and a very rare site for an NEC origin; besides, this tumor type is infrequent in the uterine cervix and bears an unfavorable prognosis when present. Therefore, when encountering a high-grade metastatic tumor within the thyroid, an NEC has to be considered in the differential diagnosis for a prompt diagnosis and an appropriate treatment.

## Introduction

The neuroendocrine (NE) system consists of a glandular system (the pituitary, the parathyroids, and the NE adrenal) and a diffuse system (scattered cells in the thyroid, pancreas, digestive and respiratory tracts, the skin, and urogenital system). Neuroendocrine tumors (NETs) are a heterogeneous group of tumors originating in these locations but the gastrointestinal tract and lungs are the most common primary tumor sites. Less common sites of origin are the thyroid, skin, pituitary, adrenal, and genitourinary tract, whereas, in females, the most frequent site is the cervix. The majority of NETs are sporadic and are associated with multiple endocrine neoplasia type 1 (MEN-1), MEN-2, von Hippel-Lindau syndrome, neurofibromatosis, and tuberous sclerosis [1-4].

NETs of the thyroid are rare entities, and one of the foremost important subgroups includes those derived from thyroid parafollicular cells, also called C cells, which are C-cell hyperplasia and medullary carcinoma. Further, its variant, medullary thyroid carcinoma (MTC) constitutes about 10% of all malignant tumors of the thyroid [5-7]. The histologic appearance of MTC varies and usually consists of sheets and solid nests of round cells with granular eosinophilic cytoplasm and a prominent eccentric nucleus with evenly dispersed chromatin. In some cases, the tumor cells can exhibit marked nuclear pleomorphism, a prominent central nucleolus, and eosinophilic granular cytoplasm, resembling Hürthle cell tumors, while, in other cases, the tumor cells can show prominent nuclear grooves and even inclusions, which can be mistaken for PTC, the most common histological subtype of thyroid carcinoma [6,8].

Interestingly, metastatic NETs to the thyroid can also mimic a primary NET of the thyroid, especially MTC. The incidence of metastases to the thyroid is low, occurring in malignant lymphoma, carcinomas of the breast, lung and kidney, head and neck tumors, and gastrointestinal tract malignancies; however, the most common origins of metastatic NET to the thyroid are lung, larynx, and gastrointestinal tract [1,4,5,9-13]. The differentiation of metastatic disease from MTC can be usually seen with an immunohistochemical examination; secondary NETs of the thyroid usually are negative for calcitonin and carcinoembryonic antigen (CEA). The management of these types of tumors creates a big challenge for physicians because of the different clinical presentations and varying degrees of aggressiveness they possess [6].

## Case presentation

**Figure 1:**
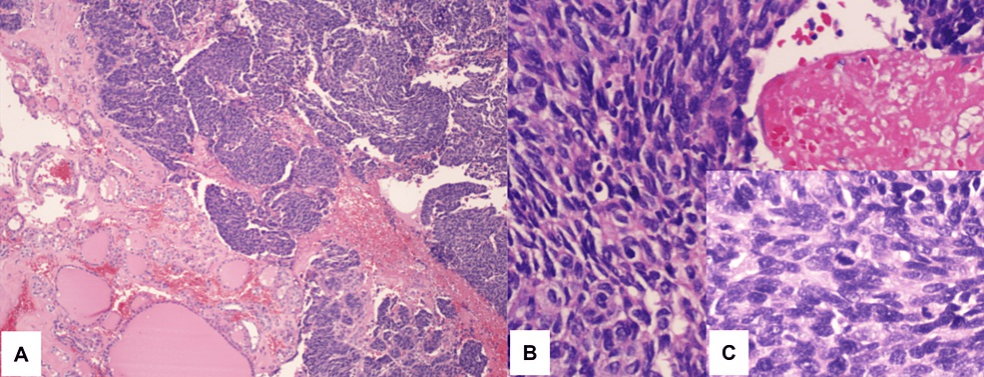
Tumor in the thyroid gland. (A) Panoramic picture of the tumor in the thyroid gland (H&E, 40×); (B) poorly differentiated tumor cells with necrosis (H&E, 200×); (C) insert with frequent mitoses (H&E, 400×). H&E: hematoxylin and eosin

Our patient is a 48-year-old lady with a medical history of diabetes mellitus type two, essential hypertension, and recent history of cervical cancer stage IIIB, diagnosed at another institution. The original diagnosis, based on a cervical biopsy, was reported as a high-grade, poorly differentiated carcinoma, negative for estrogen receptor (ER), progesterone receptor (PR), and p40, while positive for p16 and vimentin, suggestive of an endocervical adenocarcinoma of possible endometrial type. One month after the cervical biopsy was performed, the patient underwent a whole-body positron emission tomography/computed tomography (PET/CT) scan, which showed a hypermetabolic cervical mass measuring 4.4 × 5.6 × 3.9 cm with standardized uptake values (SUVs) peaking at 7.9, consistent with known cancer there; a right paracervical lymph node measuring 1.3 cm with SUVs peaking at 3.9, with concerns of being metastatic; and a multinodular thyroid goiter with mild uptake in the mid-left thyroid nodules with SUVs peaking at 3.3 and the largest measuring 3.5 × 2.4 cm, concerning for possible malignancy. The patient was scheduled for a thyroid ultrasound three weeks later, in which the radiologic impression turned out to be a multinodular goiter. The most suspicious nodules were one on the right lobe measuring 2.9 cm, TR3, and one on the left lobe measuring 4.5 cm, TR3. A month later, she underwent a left thyroid gland nodule fine-needle aspiration (FNA), also in a different institution, and was diagnosed as PTC, Bethesda VI. The patient presented to our hospital two months after the diagnosis of PTC, complaining of discomfort with head movements and difficulty when lying down, for which a total thyroidectomy and direct laryngoscopy were scheduled. The patient tolerated the procedure very well. On larynx evaluation, the bilateral vocal cords were noted to be mobile. After the patient awakened, she was sent to the recovery room in good condition. Per the pathology department, nodular hyperplasia of the thyroid with focal dystrophic calcification was found, together with several metastatic high-grade carcinoma foci in both thyroid lobes ranging from 0.1 to 0.5 cm (Figure 1).

**Figure 2:**
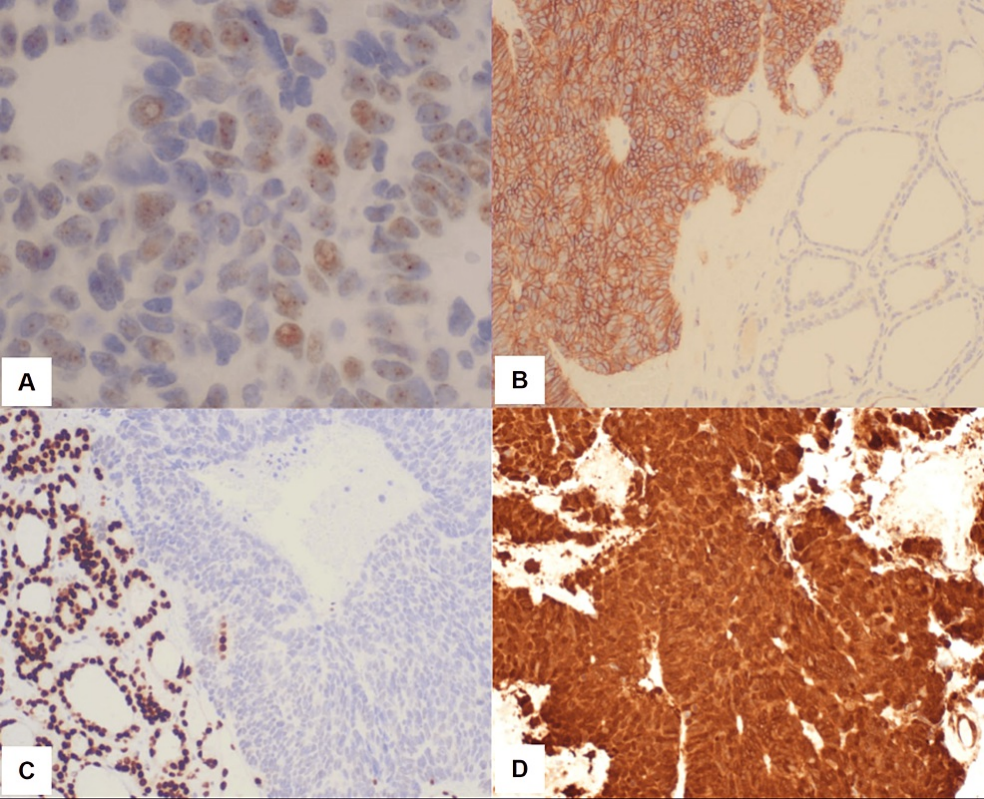
Immunohistochemistry of tumor cells. (A) ER positive (400×); (B) synaptophysin positive (100×); (C) TTF-1 negative (100×); (D) p16 positive (100×). ER: estrogen receptor; TTF1: thyroid transcription factor 1

On immunohistochemistry (IHC), tumor cells were positive for CD56, cytokeratin (CK) 7, CK20, p16, synaptophysin; partially positive for ER and CEA; and negative for CK5/6, thyroid transcription factor 1 (TTF-1), and p63 (Figure 2). Based on the overall primary and metastatic tumor morphology and IHC markers, a final diagnosis of metastatic neuroendocrine carcinoma of the cervix (NECC) to the thyroid was established.

## Discussion

Squamous cell carcinoma (SCC) is the most common histologic subtype of cervical cancer, accounting for approximately 80% of cases; the second one is cervical adenocarcinoma, accounting for about 15% of cervical cancer cases. Adenosquamous and NECC are rare cervical tumors that account for the remaining 5% of the cases. In addition, NECC is more aggressive than SCC or adenocarcinoma and has a poorer prognosis [14,15]. NET of the uterine cervix (NTUC) is a rare kind of malignant cervical tumor. There are four categories of NTUC, namely, small-cell NEC (the most common type of NTUC), large-cell NEC, atypical carcinoid tumor, and typical carcinoid tumor (almost non-existent in the cervix). Small-cell NEC includes no more than 2% of all invasive cervical cancers, and the mean annual incidence is 0.06 per 100,000 women, compared with 6.6 for SCC and 1.2 for adenocarcinoma [15-18].

Our case refers to a NECC with thyroid metastasis that was initially misclassified as a high-grade adenocarcinoma. At first, it was thought that our patient’s diagnosis was a high-grade poorly differentiated adenocarcinoma, highly suggestive of human papillomavirus (HPV)-associated endocervical adenocarcinoma with positive IHC for p16, negative for ER/PR; however, due to vimentin positivity, a possible endometrial type was thought to be present; cervical tumor cells were also negative for p40, which excluded an SCC. Pathologically, NECC is difficult to differentiate from other cervical malignancies, and histological examination and IHC have a crucial role in the diagnosis. The morphological distinction between NECC and poorly differentiated SCC is challenging, both are known to be related to HPV infection. p16 represents a reliable surrogate marker for the infection. A current study revealed that CD56 may be the most sensitive but not a special marker for diagnosis of small-cell NEC; moreover, IHC for p63 and p40 may also be useful because these markers are consistently expressed in SCC. Cervical adenocarcinoma is the major differential diagnosis for NECC, it is classified into HPV-related, endometrioid, clear-cell, and intestinal types. On IHC, HPV-associated adenocarcinoma tumor cells are negative for ER and PR and positive for p16; endometrioid adenocarcinoma of endometrial origin tumor cells are negative for ER, PR, and p40 and positive for p16 and vimentin. The one marker that is useful to differentiate them from NECC is CD56, which is negative in adenocarcinoma of the cervix but expresses in NECC [2,15].

On further follow-up studies, our patient was found to have suspicious thyroid nodules and underwent an FNA, which was diagnosed as PTC on morphology. Metastatic NETs from the lung, larynx, and gastrointestinal tract to the thyroid can mimic primary NETs of the thyroid, especially MTC, which has been reported to histologically show prominent nuclear grooves and even inclusions and can be mistaken for PTC [6].

With the previous diagnosis of PTC, our patient underwent a total thyroidectomy. In addition to the nodular hyperplasia, a metastatic poorly differentiated carcinoma with NE features was found within the thyroid without evidence of PTC. Thyroid metastases are not common even though the thyroid gland has a rich vascular supply. The primary carcinomas that metastasize to the thyroid originate from the kidney, gastrointestinal tract, lung, and breast; other types such as melanoma, sarcoma, and pleura mesothelioma have also been reported. Although NECC has a tendency for metastasis to the lung, liver, brain, bone, pancreas, kidney, breast, spleen, adrenal gland, and lymph nodes, in our research, we only found one reported case of NECC metastasizing to the thyroid in an FNA sample [10,15,17,18].

Table 1 shows a comparison of the immunophenotypic features of the most common cervical malignancies, PTC, and NEC. There are similarities between our case and other differential diagnoses, especially with SCC; however, due to the patient’s history, and positive IHC for CD56, CEA, p16, synaptophysin, CK7, CK20, and ER, and negative for p63, CK5/6, and TTF1, we justify our final diagnosis of thyroid metastasis from a primary NECC.

**Table 1:** Immunohistochemical markers in the differential diagnosis of neuroendocrine carcinoma. N/D: not done; CK5/6: cytokeratin 5/6; TTF-1: thyroid transcription factor 1; ER: estrogen receptor; CEA: carcinoembryonic antigen

	Metastatic neuroendocrine carcinoma of the thyroid (our case)	Neuroendocrine carcinoma of the cervix (outside biopsy)	Neuroendocrine carcinoma of the cervix (literature)	Papillary thyroid carcinoma	Squamous cell carcinoma	Adenocarcinoma of the cervix	Adenocarcinoma of the endometrium
p16	+	+	+	-	+	+	-/ Rare +
CD56	+	N/D	+	-	-	-	-
Vimentin	N/D	+	-/ Rare +	+/-	+/-	-	+
p40	N/D	-	-	-	+	-	-
p63/CK5/6	-	-	-/ Focal +	-	+	-	-
TTF-1	-	N/D	-/+	+	-/+	-	-
ER	Partial +	-	+/-	-	+/-	-	+
CEA	Partial +	N/D	-/+	-	-/+	+	-/+

## Conclusions

NECC is a rare variant of cervical cancer with a poor prognosis. In patients with NETs, metastatic disease to the thyroid is uncommon. We only found one reported case of NECC metastasizing to the thyroid in an FNA sample. The thyroid examination is not highlighted in patients with a previous history of malignancy of the cervix. Our case demonstrates that they must be considered for metastatic disease despite not presenting palpable nodules, swelling, or thyroid enlargement. FNA provides a quick, easy, and reliable way of diagnosis if the sample is sufficient; otherwise, the diagnosis relies on the examination, histological examination, and especially on IHC.
